# Establishment and characterization of HROC69 – a Crohn´s related colonic carcinoma cell line and its matched patient-derived xenograft

**DOI:** 10.1038/srep24671

**Published:** 2016-04-18

**Authors:** Florian Kuehn, Christina S. Mullins, Mathias Krohn, Christine Harnack, Robert Ramer, Oliver H. Krämer, Ernst Klar, Maja Huehns, Michael Linnebacher

**Affiliations:** 1 University Medicine Rostock, Department of General-, Thoracic-, Vascular- and Transplantation Surgery, Rostock, Germany; 2 University Medicine Rostock, Department of General Surgery, Molecular Oncology and Immunotherapy, Rostock, Germany; 3 University Medicine Rostock, Institute of Toxicology and Pharmacology, Rostock, Germany; 4 University Medical Center Mainz, Department of Toxicology, Mainz, Germany; 5 University Medicine Rostock, Institute of Pathology, Rostock, Germany

## Abstract

Colitis-associated colorectal cancer (CAC) seems to be a rather unique entity and differs in its genetic alterations, tumour formation capacities, and clinical features from sporadic colorectal carcinoma. Most descriptions about tumour biology of CAC refer to ulcerative colitis; data about Crohn´s colitis related carcinomas are scarce. The majority of patients with Crohn´s disease are under immunosuppression which generates a different environment for tumour growth. We first describe the clinical case of a fast growing CAC in a long-term immunosuppressed patient with Crohn´s disease and successful establishment and characterization of carcinoma cell lines along with their corresponding patient-derived xenograft. Subsequently, these tumor models were molecularly and functionally analysed. Beside numerous chromosomal alterations, mutations in TP53, APC, PTEN and SMAD3 were identified. The cell lines express numerous cancer testis antigens, surface molecules involved in immune evasion but low levels of HLA class I molecules. They show strong invasive but in comparison weak migratory activity. The present work is the first description of patient-derived *in vitro* and *in vivo* models for CAC from a Crohn´s disease patient. They might be valuable tools for analysis of genetic and epigenetic alterations, biomarker identification, functional testing, including response prediction, and the development of specific therapeutical strategies.

Colitis-associated colorectal cancer (CAC) seems to be a rather unique tumour entity since it differs substantially with regard to genetic alterations, tumourigenesis as well as clinical features from sporadic colorectal carcinoma (CRC). Most studies on molecular alterations in CAC focused on ulcerative colitis (UC) and thus data on characterization of Crohn´s disease (CD) associated CRC are scarce. Patients with UC are known to be at increased risk of developing CRC^1^,^2^,^3^,^4^,^5^. Contrary, there is still controversy about cancer risk in CD. However, subgroup analyses and population-based studies for longstanding Crohn´s colitis have shown a similar risk for developing CRC^6^,^7^,^8^,^9^.

Recently, the groups of Renz and Scaringi could show lower 5-year survival and higher local recurrence rates in inflammatory bowel disease (IBD)-related compared to sporadic CRC and suggest a link to the differences in tumour biology^10^,^11^.

A likely explanation could come from the fact that IBD patients frequently receive immunosuppressive treatment for long time periods. This has been acknowledged as risk factor for skin cancer, lymphoma and acute myeloid leukemia^12^,^13^. However, the immunological reasons for cancer formation and tumour progression in strongly immunosuppressed patients with IBD have not been examined yet. It is obvious that inhibition of immune effector cells participating in recognition and destruction of cancer cells leads to a decreased immunosurveillance^14^. Moreover, it is now well established that immunogenicity of a tumour increases with its mutational load. In this context, we hypothesize that mutations in (pre)malignant cells are better tolerated in acute and chronically immunosuppressed patients. This does not necessarily lead to tumour formation but such a “tumour-friendly” environment will dramatically accelerate tumour growth after initial cancer manifestation.

Here, we first describe the clinical case of an extremely fast growing CAC in a long-term immunosuppressed patient with CD and second, the establishment and characterization of (a) cancer cell line(s) and the corresponding patient-derived xenograft (PDX) from this tumour. To the best of our knowledge, this is the first report on successful CAC cell line establishment.

## Methods

### Tumour Preparation, Xenografting & Cell Line Establishment

The resection specimen was received fresh from surgery and the tumour sample was processed immediately. For cryopreservation and subsequent xenografting, pieces of 3 × 3 × 3 mm were vitally frozen (FCS, 10% DMSO) at −80 °C. Further samples were snap frozen in liquid nitrogen and stored in the gas phase above liquid nitrogen for molecular analysis.

Cell culture was started from a single cell suspension, by mechanically dissecting a small tumour piece (crossed scalpels) and passing through a cell strainer (100 μm). Cell suspension was seeded on collagen-coated plates using modified DMEM/Ham’s F12 (1:1) medium (+10% FCS, 2 mM L-glutamine, supplements, antibiotics and antimycotics) and incubated at 37 °C in a humidified atmosphere of 5% C0_2_. All cell culture reagents were obtained from Pan Biotech (Aidenbach, Germany), antibiotics and antifungal agents were provided by the university hospital’s pharmacy. Medium was changed regularly. Initial passage into a 25 cm^2^ culture flask was performed when substantial tumour cell growth was observed. Continually growing cell cultures were further passaged and regularly stocked in low passages. A B-lymphoid cell line (B-LCL) was generated from purified peripheral blood leukocytes of the patient by Epstein-Barr virus (EBV)-transformation as described previously^15^. Outgrowing B-LCLs were harvested, expanded, characterized, and frozen.

For *in vivo* engraftment, six-week-old female NMRI nu/nu mice were used as recipients. Mice were bred in the university’s animal facility and maintained in specified pathogen-free conditions. All surgical interventions were performed under Ketamin/Xylazin anaesthesia (dose: 90/25 mg/kg body weight), and all efforts were made to minimize suffering. Subcutaneous tumour implantation was performed as previously described^16^. Established xenografts (>1.500 mm^3^) were removed and underwent *in vitro* culture protocols as described above.

All procedures involving patient material were approved by the Ethics Committee of the Medical faculty, University of Rostock (reference number II HV 43/2004) in accordance with generally accepted guidelines for the use of human material. Informed patient consent was obtained in written. All *in vivo* experimental procedures were carried out in strict accordance with the recommendations in the Guide for the Care and Use of Laboratory Animals of the National Institutes of Health. The protocol was approved by the Committee on the Ethics of Animal Experiments of the University of Rostock (Landesamt für Landwirtschaft, Lebensmittelsicherheit und Fischerei Mecklenburg- Vorpommern; Thierfelder Str. 18, 18059 Rostock, Germany; permit number: LALLF M-V/TSD/7221.3-1.1-071-10).

### Cell culture

The CRC cell lines HROC59, HROC60, HROC80 T1 M1 and HCT116 were cultured in T75 culture flasks using DMEM/Ham’s F12 medium supplemented with 10% FCS and 2 mM L-glutamine as described before^17^.

### Histology and Immunohistochemistry of Original Tumours

Histopathological examination of primary tumours was done according to standard protocols for clinicopathological CRC staging as well as additional staging information was compiled from patients’ clinical charts. H&E sections; b-catenin, MLH1, and MSH2 immunostainings were obtained from paraffin-embedded tumours.

### Molecular Analysis

Molecular classification was done as previously described^18^. Mismatch repair deficiency was examined using the Bethesda panel and additionally the mononucleotide marker Cat25. Mutational analyses for APC, TP53, KRAS and BRAF genes were performed. Additionally, mutations in SMAD2, SMAD3, SMAD4 and the TGFß receptor type 2 were screened using a targeted sequencing approach (Ion AmpliSeq™ Technology of Thermo Fisher Scientific, Waltham, USA). Finally, DNA-methylation in CIMP-sensitive promoters was traced by the MethyLight technology with a modified marker panel^19^. Chromosomal instability was analysed using the Human SNP Array 6.0 and gene expression was assessed using the Human Genome U133 Plus 2.0 Array (in comparison to Arrays for normal colon tissue from Gene Expression Omnibus: GSM1014798_AFX_1_N1, GSM1014799_AFX_1_N2, GSM1014800_AFX_1_N3) from Affymetrix (Cleveland, USA) according to manufacturer’s instructions. Identity testing was performed through short tandem repeats analysis at 9 different loci (D5S818, D13S317, D7S820, D16S539, vWA, TH01, TPOX, CSF1PO and amelogenin for gender determination) by multiplex PCR. Amplicons were separated by capillary electrophoresis and detected on an automated ABI 3500 Genetic Analyzer and analysed using GeneMapperID software (Thermo Fisher Scientific, USA).

### *In vitro* Growth Kinetics

Cells (5 × 10^5^ cells) were plated in 5 ml media in quintuplicate T25 culture flasks and allowed to attach for 48 h; vital cells were assessed by trypan blue staining and one flask was counted every 24 h for five consecutive days using a Neubauer chamber.

### Flow Cytometry

Cell surface marker expression on established tumour cell lines was traced by flow cytometry with and without IFN-γ pre-treatment as described in detail before using a panel of fluorescent antibodies (anti-CD15 (Lewis X)-PE, anti-CD26-PE, anti-CD44-FITC, anti-CD54-APC, anti-CD66acde-FITC, anti-CD95-FITC, anti-CD178 (CD95L)-PE, anti-CD274 (PD-L1)-APC, anti-CD276 (B7-H3)-PE, anti-CD279 (PD-1)-PerCP/Cy5.5, anti-CD326-APC, anti-HLA-I-FITC, anti-HLA-II-FITC, anti-HLA-E-PE, anti-HLA-G-APC, anti-human IgA-APC, anti-human IgG-FITC, anti-human IgM-APC, and anti-IDO (Indolamin-2,3-Dioxygenase)-PE)^20^. Samples were analysed using CellDiva software (BD Biosciences, Heidelberg, Germany).

### Western blot

Western blot was done as previously described^21^. Antibodies specific for the following targets were from Santa Cruz (Heidelberg, Germany): BAX, β-catenin, HSP90, histone deacetylase (HDAC)2, PIG3, p53 and p21. Anti-survivin was obtained from Novus Biologicals (Cambridge, UK).

### Migration and Invasion Assay

Tumour cell invasion was examined using a classical Boyden chamber test (8 μm pore size in a 24-well plate format) with Matrigel-coating (BD Biosciences) according to the manufacturer’s instructions. Cells were suspended to yield a cell number of 2 × 10^5^ cells per upper Boyden chamber in 500 μl serum-free medium. Medium in the lower Boyden chamber was supplemented with 10% heat-inactivated FCS serving as chemo-attractant. Following a 72 h incubation period, the non-invading cells on the upper surface of the inserts were removed with a cotton swab, and viability of cells on the lower surface was measured by the colorimetric 4-[3-(4-iodophenyl)-2-(4-nitrophenyl)-2H-5-tetrazolio]-1,6-benzene disulfonate (WST-1) test (Roche Diagnostics, Mannheim, Germany)^22^. Quantification of migration was performed in parallel using the same protocol but with uncoated upper Boyden chambers.

### *In vitro* drug response

Cells (1 × 10^5^ cells) were plated in 150 μl media (DMEM/Ham’s F12 supplemented with 10% FCS, 2 mm L-glutamine and antibiotics) per well in triplicate in 96-well flat bottom culture plates and allowed to attach for 24 h. The following concentration ranges of drugs were tested (given are final concentrations in the experimental wells): (1) 0.4 μM–6.7 mM 5-FU (Medac, Wedel, Germany), (2) 244 nM–1 mM Irinotecan (Pfizer, Berlin, Germany), (3) 30 nM–30 μM Cisplatin (Teva GmbH, Ulm, Germany), (4) 4.7 nM–300 nM Gemcitabine (Fresenius Kabi, Bad Homburg, Germany), (5) 30 nM–30 μM Rapamycin (Pfizer), (6) 0.9 nM–60 nM Taxol (Medac), (7) 128 nM–2 mM Temodal (Sigma-Aldrich, Taufkirchen, Germany), (8) 32 nM–500 μM BCNU (Bristol-Myers Squibb, New York, USA), (9) 32 nM–500 μM CCNU (Sigma-Aldrich) and the therapeutic antibodies Bevacizumab (Roche, Basel, Switzerland), Cetuximab (Bristol-Myers Squibb) and Rituximab (Roche) all in the range of 313 ng/ml–20 μg/ml. Equal volumes DMSO (for cells treated with Cisplatin, Rapamycin, Temodal, BCNU and CCNU) were added to cells serving as untreated controls. Cells were incubated with the substances for 72 h, and media were replaced together with substances in the same concentrations as before. After a second 72 h incubation period cell viability was assessed by a crystal violet assay. Viability was calculated as percentage of live cells compared to the untreated control cells.

### Statistics

In invasion and migration tests comparisons between groups were performed with ANOVA plus post hoc Bonferroni test using GraphPad Prism 5.0 (GraphPad Software, San Diego, CA, USA). Results were considered to be statistically significant at values of P < 0.05.

## Results

### Clinical case of a CD-associated carcinoma after long-term immunosuppression

**Figure 1:**
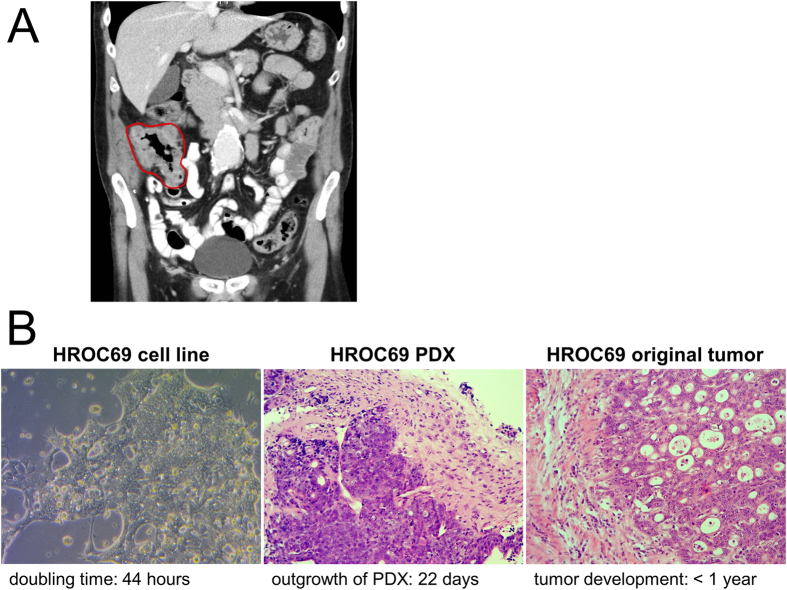
Patient tumour and model imaging. (**A**) CT-scan showing the large tumour affecting cecum and ascending colon (red surrounding)–without proof of distant metastases. (**B**) micro-photographs (20×) of the patient-derived cell line HROC69, an H&E stain of HROC69 PDX and an H&E stain of patient HROC69 tumour. The time for tumour (model) growth is indicated below each image.

We report on a 62 year old patient with longstanding CD of the colon and fistulising rectum manifestation. First diagnosis of CD was at patient’s age of 42 years. Because of complicated fistulising rectal disease, the patient was treated with a combination of azathioprine and the TNF-α-antibody infliximab for 9 years. Surgical consultations and interventions for abscess formation or fistula also took place. Azathioprine was applied daily according to body weight. Additionally, the patient received infliximab infusions every 8 weeks (52 infusions in total). In a surveillance colonoscopy a large carcinoma affecting cecum and ascending colon was detected. Crohn´s luminal activity was mild and mostly restricted to the rectum. One year before, the patient underwent surveillance pan-colonoscopy by the same physician showing rectal and perianal CD but no signs of malignant transformation. Luminal colonic disease was described as non-severe and mostly in remission. After completing diagnostics without proof of distal metastases (Fig. 1A), tumour resection was conducted by a right-sided hemicolectomy.

Macroscopically, the tumour size was 12 × 8 × 1.5 cm, reaching from an infiltrated terminal ileum to the ascending colon. Microscopically, an adenocarcinoma with loss of differentiation, invasion of pericolonic fatty tissue and lymphatic tract invasion but without involvement of 34 resected lymph nodes was revealed (UICC stage II; G3; pT3 pN0 (0/34) L1 V0 R0). Due to a complicated postoperative course with multiple surgical revisions, patient HROC69 did not receive adjuvant therapy. He died of an aortic aneurysm 2 years after surgery for his CAC.

### Establishment of permanent cell lines and a PDX model

Patient-derived primary tumour model establishment was attempted in parallel *in vitro* (patient-derived cell line) and *in vivo* (PDX). Outgrowth of cells in culture occurred immediately (which is a very rare event for primary CRC cultures). These outgrowing cells had the typical colon epithelial morphology and (after several passages) cells doubled every 44 hours (Fig. 1B).

Similarly to *in vitro*, substantial tumour formation *in vivo* in immunodeficient NMRI nu/nu mice could be observed already 22 days after initial patient tumour engraftment (tumour volume of four engrafted flanks was 245.9 mm^3^ ± 32.0). Histological comparison of the original patient tumour and the corresponding PDX revealed the conservation of tissue architecture in the model (Fig. 1B); both the primary patient tumour and the PDX showed low budding and were negative for b-Kat (data not shown). A human specific PCR (amplification of the human-specific mitochondrial cytochrome b region) confirmed human identity of the PDX (data not shown).

**Figure 2:**
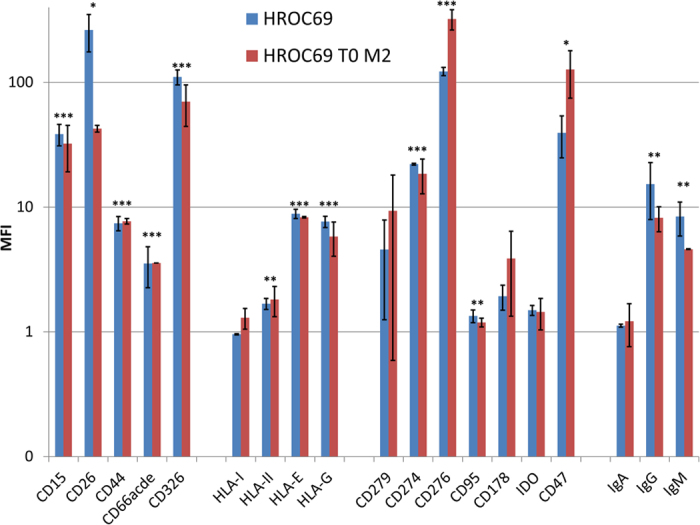
Expression of HLA molecules, immune checkpoint proteins and CRC markers. Expression of epithelial tumour markers (CD15, CD26, CD44, CD66acde and CD326), HLA molecules (HLA-I, HLA-II, HLA-E and HLA-G), immune checkpoints (CD279, CD274, CD276, CD95, CD178, IDO and CD47) and immunoglobulins (IgA, IgG and IgM) was assessed by flow cytometry using a BD FACSARIA II. Mean fluorescent intensity (MFI = (fluorescence intensity of antibody staining-fluorescence intensity of control)/ fluorescence intensity of control) ± SEM of n = 3 analyses are represented in the bar chart for the patient-derived cell line (HROC69, blue) and the PDX-derived cell line (HROC69 T0 M2, red).

**Figure 3:**
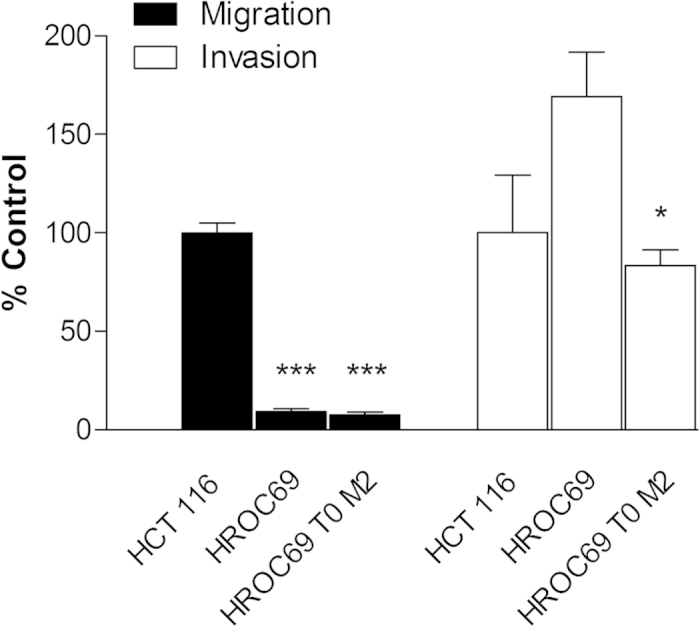
Migratory potential and invasiveness of HROC69 cells. Invasion and migration of the PDX-derived cell line HROC69 T0 M2 and the patient-derived cell line HROC69 in comparison to the reference cell line HCT116 was analysed. Cells were subjected to migration assay (Migration, black bars) and matrigel invasion assay (Invasion, white bars). For invasion and migration assays, cells were used at a density of 2 × 10^5^ cells per upper Boyden chamber in a 24-well-plate format in 500 μl serum-free DMEM, respectively. In Boyden chamber assays DMEM containing 10% FCS was used as chemoattractant in the companion plate. Subsequently, cells were incubated for 72 hours. Values are means ± SEM of n = 4. ***P < 0.001, vs. HCT116 FL of the respective assay, ANOVA plus post hoc Bonferroni-test.

A cell line from the PDX could again easily be established (HROC69 T0 M2). Both the patient- and PDX-derived cell lines express moderate to high levels of common epithelial cell markers: CD66acde <CD44 < CD15 (Lewis X) <CD26 < CD326 (Fig. 2). With the exception of sialyl-lewis x (CD15), the expression did not differ from that of a sporadic CRC patient-derived cell line (Supplementary figure 1). Some of these markers alone or in variable combinations have repeatedly been described as indicating stem cell related properties^23^,^24^. Invasion and migration analyses revealed a remarkable difference in the infiltrative activity (when comparing the patient-derived cell line HROC69 and the PDX-derived cell line HROC69 T0 M2; p = 0.0254). Compared to HCT116, which has repeatedly been described as relatively highly-invasive, the PDX-derived HROC69 T0 M2 was slightly less invasive whereas the patient-derived HROC69 tended to be slightly more invasive (Fig. 3). Both PDX-derived HROC69 T0 M2 and the patient-derived HROC69 revealed significantly less migratory activity (through uncoated Boyden chambers) than the HCT116 cell line. The fact that the HROC69 cell lines are relatively immobile but highly invasive implies that they must be very active secretors of proteolytic enzymes.

To confirm that established models derived from the patient presenting with the Crohn´s related CRC, identity testing was performed and genomic finger print profiles of the PDX, the HROC69 cell lines and the HROC69 Bc as normal tissue control were compared and revealed no differences (Supplementary table 1).

### HROC69 has several chromosomal aberrations, mutations in APC, TP53, PTEN and SMAD3 and expresses numerous cancer testis antigens

Molecular analyses for common features of CRC such as the mutation status (of the genes APC, TP53, PTEN, KRAS and BRAF), methylation degree of CpG islands and stability of microsatellite regions were performed on the models in comparison to the original tumour material (Table 1). Of note, all mutations and molecular characteristics found in the original patient tumour were maintained in the tumour models. The tumour and corresponding models showed a high degree of aneuploidy, had mutant APC (R1450*), TP53 (R306*) and PTEN (E7*, R130Q, R142Q) genes, but normal levels of CpG island methylation and microsatellite regions appeared stable. In accordance with this molecular profile and spontaneous occurrence of the tumour, it was classified as a sporadic standard CRC according to Ostwald and co-workers^18^. Additionally, mutations in the genes of the TGFß pathway (SMAD2, SMAD3, SMAD4 and TGFß receptor type II) have recently been described for CRC^25^.Thus, we screened for mutations in these genes as well; using a targeted sequencing approach. In all CAC tumour models, a mutation in the SMAD3 gene (Ala382Val) was found which was not present in the patient´s germline DNA (Table 2).

Larger genomic aberrations, single nucleotide polymorphisms and general gene expression were assessed by the Affymetrix microarrays (I) Human SNP Array 6.0 and (II) Human Genome U133 Plus 2.0 Array. While the chromosomes of the B-LCL line HROC69 Bc show no obvious changes, both HROC69 CAC models, the patient-derived cell line and the PDX have numerous aberrations (Supplementary figure 2). In both models the tip of chromosomes 1q, 8q and 10q are amplified and 13q shows a chromosomal gain of more than half the arm. The tips of chromosome 4 as well as 8p are entirely lost. Additionally, the cell line has an amplified 5q whereas the PDX has amplifications of 10p and 14q (Supplementary figure 2).

The expression analysis revealed that of the 20 most over-expressed genes in comparison to normal colon tissue, half belong to the group of cancer-testis antigens and most of the remaining genes have described functions in regulation of gene/protein expression (Supplementary table 2). Within the 20 lowest expressed genes, at least three tumour suppressor genes were found. Most of the remaining genes are essential in cell growth control, metabolism and stress response (Supplementary table 2).

### HROC69 cell lines express low levels of HLA class I but relatively high levels of several immune checkpoint proteins

An important prerequisite for antigen-specific immune recognition and tumour cell elimination is the sufficient expression of HLA molecules, in especially class I. In both HROC69 cell lines, HLA class I was barely expressed (Fig. 2) and even IFNγ pre-treatment did not increase expression. The likewise weak to absent expression of HLA class II was slightly increased after IFNγ incubation (data not shown). Contrarily to the major HLA molecules, HROC69 and HROC69 T0 M2 express higher levels of HLA-E and -G molecules, involved in immune suppression (Fig. 2).

Additional immune checkpoint proteins and regulators of immune responses were analysed by flow cytometry. Again, results obtained with the patient-derived cell line HROC69 matched those of the PDX-derived cell line HROC69 T0 M2: both cell lines had low to no expression of IDO, CD95, CD36 (FAT), CD73 (5′NT) and CD273 (PD-L2) (Fig. 2 and some data not shown). Whereas, expression levels of CD276 (B7-H3), CD47 (IAP) and CD274 (PD-L1) were rather high (Fig. 2). CD279 (PD-1) and CD178 (Fas-L) expression was intermediate (Fig. 2). In comparison to a classical CRC patient-derived cell line (HROC80 T1 M1) from an immunosuppression-naive patient, the CAC derived cell lines HROC69 and HROC69 T0 M2 show a more pronounced immunosuppressant phenotype. Especially a higher proportion of the CAC tumour cells express PD-L1 and B7-H3 (expressed by a variety of solid tumour cells) (Supplementary figure 3). Moreover, both HROC69 cell lines express the immunoglobulins IgG and IgM at their cell surface, but no IgA (Fig. 2).

### Assessment of p53, its target genes, and of HDAC1/HDAC2

Next, we analysed the expression of the transcription factor p53 and of some of its target genes by Western blot. These proteins are important for the regulation of the cell cycle and for apoptosis. We compared the HROC69 cell line with two other patient-derived, low-passage number CRC cell lines established in our laboratory, HROC59 (p53 wild-type) and HROC60 (p53 mutant)^17^.

**Figure 4:**
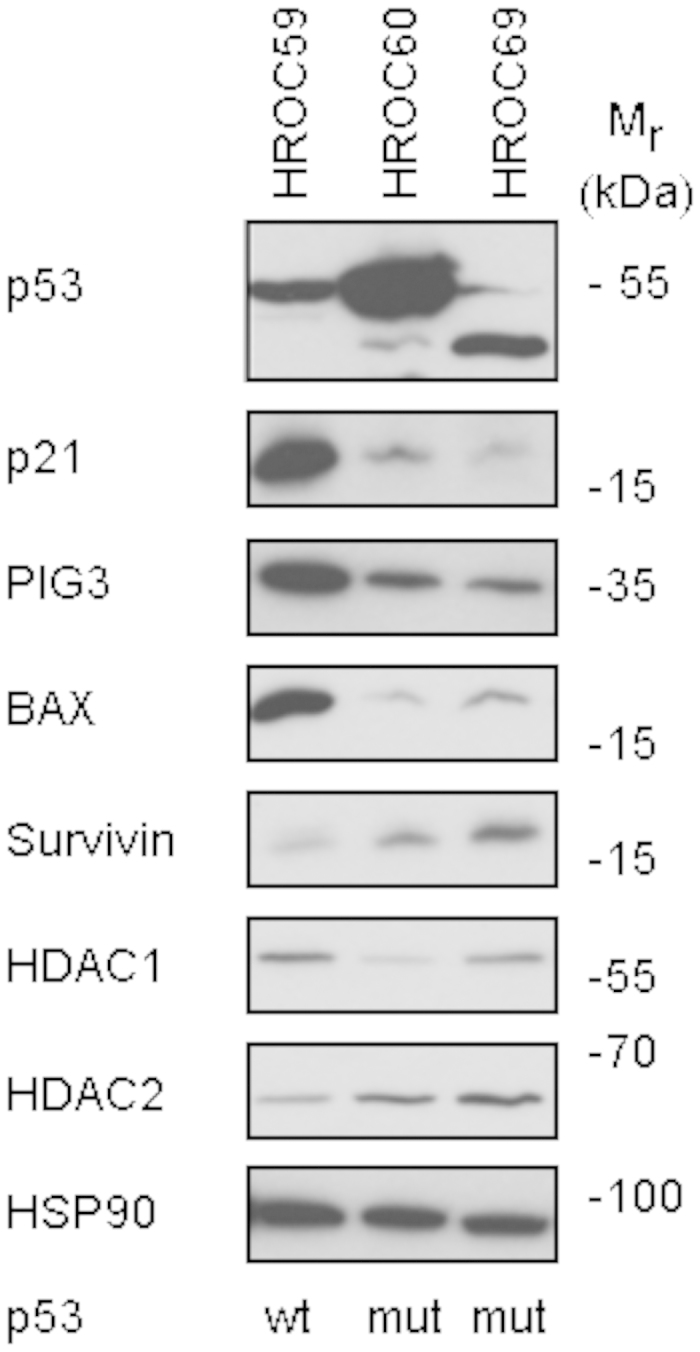
Detection of proteins involved in tumour suppression, apoptosis and stress response by immune blot. Lysates from exponentially growing cultures of the patient-derived cell lines HROC69, HROC59 and HROC60 were subjected to Western blot analysis as indicated. Expression of p53 and its target genes p21, PIG3, BAX (positively regulated) and survivin (negatively regulated), HDAC1, HDAC2, and HSP90 as the loading control were determined with specific antibodies. We used 12% gels and performed the wet transfer procedure. All antibody incubations were done overnight at 4 °C. To ensure the correct molecular weight of the proteins that we detected, we used the molecular weight standard Fermentas #SM1811.

Our data show that while the tumour suppressor protein p53 is detectable in HROC69 cells, it occurs as a faster migrating band. The levels of this shorter p53 variant were expressed at levels comparable to wild-type p53 in HROC59 and to a lower extent than mutant p53 R273H in HROC60 cells (Fig. 4).

As expected for wild-type and mutant p53, only the wild-type could induce the p53-regulated factors p21, PIG3, and BAX (Fig. 4). Likewise, the p53-repressed factor survivin was expressed at lower levels in CRC cells with wild-type p53.

Epigenetic regulators belonging to the group of HDACs can modulate p53 functions and the class I HDACs HDAC1 and HDAC2 are targets of butyrate which is abundantly present in the colonic epithelium^26^. Therefore, we analysed the expression levels of HDAC1 and HDAC2 in our cellular systems. Both enzymes were expressed at slightly different levels in all three cell lines (Fig. 4), which rules out gross alterations at the levels of these HDACs.

From these data we conclude that HROC69 possesses a short p53 protein that can most likely not exert wild-type functions.

### HROC69 cells are sensitive towards many therapeutical drugs

**Figure 5:**
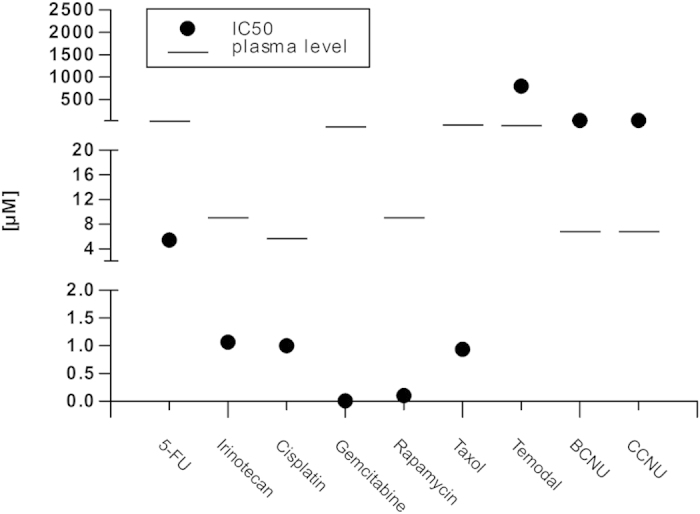
Drug response. Response to therapeutic agents is given as IC_50_ value (black dot) and clinically evident patient plasma levels are indicated by a line. Therapeutic agents used are listed on the x-axis. On the y-axis, concentrations of the therapeutic agents are displayed in μM using a segmented y-axis.

Response of the patient-derived cell line HROC69 (in passages 23–29) to therapeutics used for CRC treatment (standard of care or experimentally) was assessed in standard proliferation/cytotoxicity assays (Fig. 5). Taking achievable patient plasma levels as a reference, HROC69 cells were (highly) sensitive towards both 5-FU and Irinotecan, standard chemotherapeutics for CRC treatment. Moreover, HROC69 cells responded to Cisplatin, Taxol, Rapamycin and Gemcitabine. A response to the alkylating agents Temodal, BCNU and CCNU could only be reached using concentrations well above the achievable patient plasma levels. None of the tested therapeutic antibodies–the anti-VEGF antibody Bevacizumab and the anti-EGFR antibody Cetuximab are frequently used in CRC treatment; anti-CD20 Rituximab (in clinical use for treatment of leukaemia) served as a negative control–had any effect on cell viability even at concentration of 20 μg/ml (data not shown). In comparison to other low-passage patient-derived cell lines established in our lab, HROC69 is less sensitive to 5-FU but more sensitive to Cisplatin, Irinotecan, Gemcitabine, Taxol and Rapamycin^17^,^20^.

## Discussion

Chronic inflammatory diseases are frequently associated with an increased risk of cancer development. Patients with IBD are at risk of developing intestinal cancers via mechanisms that are only partially understood. Together with hereditary CRC syndromes, i.e. familial adenomatous polyposis and Lynch-syndrome, IBD is among the top three high-risk conditions for CRC^27^. In comparison to UC, CRC in CD has been studied less well.

For the subset of CD patients with a longstanding, anatomically substantial colitis the risk of CRC is similar to UC^8^. Indeed, a population-based study from Canada confirmed that the risk for CRC among patients with both UC and CD is approximately 2-3 fold greater than the general population^7^. A meta-analysis of various population-based studies confirmed an increased risk for CRC and small bowel cancer in patients with CD^28^.

It is still a myth that CAC are restricted to areas of grossly affected intestine^8^. However, in the Mount Sinai series as many as one third of CAC arose in distant segments of the gastrointestinal tract either far proximal or distal to macroscopically obvious CD^29^. This was confirmed in a recent study where up to 42% of CD patients developed tumours in non-inflamed mucosal areas^30^. Tumour localization of HROC69 was also in a non- or only mildly inflamed area of the colon.

Patients with CAC are significantly younger than sporadic CRC patients^31^. The patient in this report was 62 years old when cancer was diagnosed and with this not outstanding from the general risk group. An analysis of our own patient population with CD which underwent surgery between 2005 and 2014 (n = 120) revealed 6 patients with CRC. Here, mean age of the 6 patients with CRC was 56 years (unpublished data). There also is a higher rate of synchronous primary CRC in IBD patients^30^. Patient HROC69 did not show synchronous tumour formation.

The prognosis of CRC has been described to be poorer in patients with IBD^10^,^31^. Beside a higher complication rate, significantly higher local recurrence and lower 5-year-survival rates were observed for patients with IBD-related cancer.

The neoplastic transformation in IBD is thought to be similar to the adenoma-carcinoma sequence in sporadic CRC^27^. The two major pathways of colon carcinogenesis, chromosomal instability (CIN) and microsatellite instability (MSI), also apply to CAC. However, timing and frequency of these alterations differ between CAC and sporadic CRC. For example, APC loss of function, considered to be a very common early event in sporadic CRC, is much less frequent and usually occurs late in the colitis-associated dysplasia-carcinoma sequence. Conversely, TP53 mutations in sporadic neoplasia usually occur late, whereas in patients with colitis, TP53 mutations occur early and are often detected in mucosa that is non-dysplastic or indefinite for dysplasia^25^. Molecular analysis of HROC69 also shows similar chip and molecular classification data compared to our collection of sporadic carcinomas. The patient tumour and corresponding CAC models showed a high degree of aneuploidy, had mutant APC (R1450*), TP53 (R306*), PTEN (E7*, R130Q, R142Q) and SMAD3 (A382V) genes. This SMAD3 A382V mutation has, according to the COSMIC database, been observed in breast cancers but not in CRC before and is classified as potentially pathogenic with a high likelihood of a gain of function in terms of an additional phosphorylation site. Thus HROC69 might be the ideal model for functional testing of SMAD3 A382V. Moreover, there is a very high likelihood that PTEN function is completely lost in HROC69.

Our immunoblot analyses illustrate that HROC69 cells carry a truncated p53 isoform. Putative roles of this molecule will be investigated in further analyses. The same holds true for the slightly different levels of HDAC1 and HDAC2. These enzymes are targets of butyrate which is highly expressed in the colonic epithelium and it is plausible that variations in HDAC expression are an adaptation to such a milieu as well as an Achilles’ heel for treatment options^26^. Methylation becomes increasingly appreciated as a mechanism that contributes to the genetic alterations in CAC^27^. Methylation of CpG islands in several genes seems to precede dysplasia and is more widespread throughout the mucosa of UC patients^32^. However, HROC69 shows very little CpG island methylation–perhaps as a feature of CD–in contrast to UC-associated carcinoma. Similarly, MSI was also absent in HROC69.

In addition to a higher risk for developing CRC, most IBD patients receive some kind of immunosuppressive treatment during the course of their disease. Immunosuppression *per se* is a known risk factor for skin cancer, lymphoma and acute myeloid leukemia^12^,^13^. A connection between intensive immunosuppression and cancer formation as well as tumour progression in patients with IBD has not been examined so far. However, an inhibition of the innate and adaptive immune system possibly leads to a decreased immune surveillance. Besides this, immunosuppression leads to a facilitated action of oncogenic viruses and shows an increase in EBV associated malignancies, i.e. nasopharyngeal carcinoma, Burkitt´s and Hodgkin´s lymphoma^33^.

The depicted patient (HROC69) was treated with a combination of azathioprine and the TNF-α-antibody infliximab for 9 years. Nowadays, azathioprine is a widely used standard for management of chronic inflammatory diseases. Thiopurine treatment positively selects for cell variants with a defective MMR system^34^. Cells with such defects will ultimately develop MSI and express frameshift-mutated proteins. Admittedly, HROC69 shows no MSI but clear evidence for an immune evasion (HLA-I down-regulation, expression of immune-checkpoint inhibitory receptors PD-L1, IAP and B7-H3). Of note, we found an extraordinary collection of cancer-testis antigens expressed in HROC69, which is likely to evoke relatively strong antitumoural immune responses and explain the need for immune evasion.

TNF-α acts as an early tumour suppressor, thus drugs blocking TNF-α may possibly increase the risk of initial occurrence of malignancies^35^,^36^,^37^. However, the role of TNF-α inhibitors in solid malignancies is not clear. TNF-α plays an important role in natural killer/CD8 lymphocyte dependent cell lysis and in modulating adaptive immunity, an important component of tumour surveillance^38^,^39^. Therefore, it is postulated that suppressing TNF-α may enhance proliferation of solid tumors^38^,^39^. This might partially explain why a 12 × 8 x 1.5 cm bulky tumour grew in a one year time period in our patient. According to the clinical picture the *in vitro* outgrowth of cells in culture occurred immediately–which is a very rare event for primary CRC cultures [own observations].

In literature, patients treated with infliximab have a comparable frequency of neoplasia to those who never received infliximab^40^. However; most patients with IBD receive a multimodal medical therapy including more than only one biological or immunosuppressant. Considering this constellation, Scaringi *et al*. could show an increased risk for CRC in patients with IBD and extensive immunosuppression^11^.

Very similar to the clinical case described here, Peyrin-Biroulet and colleagues report on a 23 year-old patient with Crohn´s colitis who developed a bulky tumour of the cecum (pT4N2M0). Seven months prior to tumour manifestation a colonoscopy without proof of any dysplasia was conducted. In between the patient had received a total of six infliximab infusions beside azathioprine therapy. Additionally, the patient was pre-medicated with tacrolimus due to a liver transplantation three years earlier^41^.

To the best of our knowledge, this is the first description and characterization of patient-derived models for CAC from a CD patient. They might thus be of great value for identifying functionality of genetic and epigenetic alterations as well as for development of specific therapeutical strategies.

## Conclusion

CAC seems to be a rather unique entity and differs in some of its genetic alterations, tumour formation and clinical features from those of sporadic CRC. Most of the descriptions about tumour biology of CAC are referring to UC whereas data about CD related carcinoma are scarce. Additionally, the majority of patients with CD are under immunosuppression which generates a different environment for tumour escape and growth, e.g. azathioprine leading to a mismatch repair deficiency. Pre-clinical models representing molecular features of CAC are thus mandatory and provide a ready basis for subsequent preclinical evaluation of innovative treatment regimens.

## Additional Information

**How to cite this article**: Kuehn, F. *et al*. Establishment and characterization of HROC69 – a Crohn´s related colonic carcinoma cell line and its matched patient-derived xenograft. *Sci. Rep*. **6**, 24671; doi: 10.1038/srep24671 (2016).

## Supplementary Material

Supplementary Information

## Figures and Tables

**Table 1:** Molecular characterizations.

sample	PIK3CA		TP53				APC	PTEN		KRAS		BRAF	microsatellite stability						DNA-methylation						
	exon 9	exon 20	exon 5	exon 6	exon 7	exon 8		exon 1	exon 5	exon 2	exon 3	V600E	BAT25	BAT26	CAT25	D2S123	D5S346	D17S250	MSP-MLH1	PMR-MLH1	CDNK	NEUROG1	CRA	CACNA1G	MGMT
patient tumour	wt	wt	wt	wt	wt	R306*	R1450*	E7*	R142Q	wt	wt	wt	MSS	MSS	MSS	MSS	MSS	MSS	−	−	+	−	−	−	−
PDX	wt	wt	wt	wt	wt	R306*	R1450*	E7*	R130Q, R142Q	wt	wt	wt	MSS	MSS	MSS	MSS	MSS	MSS	−	−	+	−	−	−	−
HROC69 T0 M2	wt	wt	wt	wt	wt	R306*	R1450*	E7*	R130Q, R142Q	wt	wt	wt	MSS	MSS	MSS	MSS	MSS	MSS	−	−	+	−	−	−	−
HROC69	wt	wt	wt	wt	wt	R306*	R1450*	E7*	R130Q, R142Q	wt	wt	wt	MSS	MSS	MSS	MSS	MSS	MSS	−	−	+	−	−	−	−

The table lists the molecular characteristics of the original patient tumour, the PDX, the patient-derived cell line HROC69 and the PDX-derived cell line HROC69 T0 M2; including analyses of genes frequently mutated in CRC, microsatellite instability testing and the methylation status of (colon-) cancer relevant genes. wt, wild type; MSS, microsatellite stable; −, not methylated; +, methylated.

**Table 2:** TGFß pathway mutation analysis.

sample	SMAD2	SMAD3	SMAD4	TGFßRII
germline gDNA	wt	wt	wt	wt
PDX	wt	A382V	wt	wt
HROC69 T0 M2	wt	A382V	wt	wt
HROC69	wt	A382V	wt	wt

The table lists the molecular alterations of the PDX, the patient-derived cell line HROC69 and the PDX-derived cell line HROC69 T0 M2 in comparison to germline gDNA for the genes of the TGFß pathway: SMAD2, SMAD3, SMAD4 and TGFß receptor type 2. wt, wild type; TGFßRII, TGFß receptor type 2.

## References

[b1] Broström O., Löfberg R., Nordenvall B., Ost A. & Hellers G. The risk of colorectal cancer in ulcerative colitis. An epidemiologic study. Scand. J. Gastroenterol. 22, 1193–1199 (1987). 3433007 10.3109/00365528708996463

[b2] Dawson I. M. & Pryse-Davies J. The development of carcinoma of the large intestine in ulcerative colitis. Br. J. Surg. 47, 113–128 (1959). 13814581 10.1002/bjs.18004720202

[b3] Eaden J. A., Abrams K. R. & Mayberry J. F. The risk of colorectal cancer in ulcerative colitis: a meta-analysis. Gut 48, 526–535 (2001). 11247898 10.1136/gut.48.4.526 PMC1728259

[b4] Maratka Z. . Incidence of colorectal cancer in proctocolitis: a retrospective study of 959 cases over 40 years. Gut 26, 43–49 (1985). 3965367 10.1136/gut.26.1.43 PMC1432400

[b5] Lashner B. A., Silverstein M. D. & Hanauer S. B. Hazard rates for dysplasia and cancer in ulcerative colitis. Results from a surveillance program. Dig. Dis. Sci. 34, 1536–1541 (1989). 2791805 10.1007/BF01537106

[b6] Canavan C., Abrams K. R. & Mayberry J. Meta-analysis: colorectal and small bowel cancer risk in patients with Crohn’s disease. Aliment. Pharmacol. Ther. 23, 1097–1104 (2006). 16611269 10.1111/j.1365-2036.2006.02854.x

[b7] Bernstein C. N., Blanchard J. F., Kliewer E. & Wajda A. Cancer risk in patients with inflammatory bowel disease: a population-based study. Cancer 91, 854–862 (2001). 11241255 10.1002/1097-0142(20010215)91:4<854::aid-cncr1073>3.0.co;2-z

[b8] Sachar D. B. Cancer in Crohn’s disease: dispelling the myths. Gut 35, 1507–1508 (1994). 7828962 10.1136/gut.35.11.1507 PMC1375601

[b9] Ekbom A., Helmick C., Zack M. & Adami H. O. Increased risk of large-bowel cancer in Crohn’s disease with colonic involvement. Lancet 336, 357–359 (1990). 1975343 10.1016/0140-6736(90)91889-i

[b10] Renz B. W. . Clinical outcome of IBD-associated versus sporadic colorectal cancer: a matched-pair analysis. J. Gastrointest. Surg. 17, 981–990 (2013). 23475629 10.1007/s11605-013-2171-z

[b11] Scaringi S. . Colorectal cancer and Crohn’s colitis: clinical implications from 313 surgical patients. World J. Surg. 37 (4), 902–10 (2013). 23381673 10.1007/s00268-013-1922-z

[b12] Opelz G. & Henderson R. Incidence of non-Hodgkin lymphoma in kidney and heart transplant recipients. Lancet 342, 1514–1516 (1993). 7902900 10.1016/s0140-6736(05)80084-4

[b13] Gale R. P. & Opelz G. Commentary: does immune suppression increase risk of developing acute myeloid leukemia? Leukemia 26, 422–423 (2012). 21869837 10.1038/leu.2011.224

[b14] Zitvogel L., Tesniere A. & Kroemer G. Cancer despite immunosurveillance: immunoselection and immunosubversion. Nat. Rev. Immunol. 6, 715–727 (2006). 16977338 10.1038/nri1936

[b15] Maletzki C. . *Ex-vivo* clonally expanded B lymphocytes infiltrating colorectal carcinoma are of mature immunophenotype and produce functional IgG. PLos One 7 (2), e32639 (2012). 22393427 10.1371/journal.pone.0032639 PMC3290587

[b16] Linnebacher M. . Cryopreservation of human colorectal carcinomas prior to xenografting. BMC Cancer 10, 362 (2010). 20615215 10.1186/1471-2407-10-362 PMC2910693

[b17] Maletzki C. . Functional Characterization and Drug Response of Freshly Established Patient-Derived Tumor Models with CpG Island Methylator Phenotype. PLos One. 10 (11), e0143194 (2015). 26618628 10.1371/journal.pone.0143194 PMC4664421

[b18] Ostwald C., Linnebacher M., Weirich V. & Prall F. Chromosomally and microsatellite stable colorectal carcinomas without the CpG island methylator phenotype in a molecular classification. Int. J. Oncol. 35, 321–3272009 (2009). 19578746

[b19] Ogino S. . CpG island methylator phenotype, microsatellite instability, BRAF mutation and clinical outcome in colon cancer. Gut 58, 90–96 (2009). 18832519 10.1136/gut.2008.155473 PMC2679586

[b20] Maletzki C. . Establishment and characterization of cell lines from chromosomal instable colorectal cancer. World J. Gastroenterol. 21, 164–176 (2015). 25574089 10.3748/wjg.v21.i1.164 PMC4284332

[b21] Buchwald M. . SIAH ubiquitin ligases target the nonreceptor tyrosine kinase ACK1 for ubiquitinylation and proteasomal degradation. Oncogene 32, 4913–4920 (2013). 23208506 10.1038/onc.2012.515 PMC3731393

[b22] Ramer R. & Hinz B. Inhibition of cancer cell invasion by cannabinoids via increased expression of tissue inhibitor of matrix metalloproteinases-1. J. Natl. Cancer Inst. 100, 59–69 (2008). 18159069 10.1093/jnci/djm268

[b23] Belov L., Zhou J. & Christopherson R. I. Cell surface markers in colorectal cancer prognosis. Int. J. Mol. Sci. 12, 78–113 (2010). 21339979 10.3390/ijms12010078 PMC3039945

[b24] Pang R. . A subpopulation of CD26 + cancer stem cells with metastatic capacity in human colorectal cancer. Cell Stem Cell 6, 603–615 (2010). 20569697 10.1016/j.stem.2010.04.001

[b25] Krämer O. H. HDAC2: a critical factor in health and disease. Trends Pharmacol. Sci. 30 (12), 647–55 (2009). 19892411 10.1016/j.tips.2009.09.007

[b26] Fleming N. I. . SMAD2, SMAD3 and SMAD4 mutations in colorectal cancer. Cancer Res. 73 (2), 725–35 (2013). 23139211 10.1158/0008-5472.CAN-12-2706

[b27] Xie J. & Itzkowitz S. H. Cancer in inflammatory bowel disease. World J. Gastroenterol. 14, 378–389 (2008). 18200660 10.3748/wjg.14.378 PMC2679126

[b28] Jess T., Gamborg M., Matzen P., Munkholm P. & Sørensen T. I. Increased risk of intestinal cancer in Crohn’s disease: a meta-analysis of population-based cohort studies. Am. J. Gastroenterol. 100, 2724–2729 (2005). 16393226 10.1111/j.1572-0241.2005.00287.x

[b29] Greenstein A. J., Sachar D. B., Smith H., Janowitz H. D. & Aufses A. H. Jr. Patterns of neoplasia in Crohn’s disease and ulcerative colitis. Cancer 46, 403–407 (1980). 7388778 10.1002/1097-0142(19800715)46:2<403::aid-cncr2820460232>3.0.co;2-6

[b30] Bressenot A., Cahn V., Danese S. & Peyrin-Biroulet L. Microscopic features of colorectal neoplasia in inflammatory bowel diseases. World J. Gastroenterol. 20, 3164–72 (2014). 24696602 10.3748/wjg.v20.i12.3164 PMC3964388

[b31] Peyrin-Biroulet L. . Colorectal cancer in inflammatory bowel diseases: a population-based study (1976–2008). Inflamm. Bowel Dis. 18, 2247–2251 (2012). 22467511 10.1002/ibd.22935

[b32] Issa J. P., Ahuja N., Toyota M., Bronner M. P. & Brentnall T. A. Accelerated age-related CpG island methylation in ulcerative colitis. Cancer Res. 61, 3573–3577 (2001). 11325821

[b33] Münz C. & Moormann A. Immune escape by Epstein-Barr virus associated malignancies. Semin. Cancer Biol. 18, 381–387 (2008). 18996483 10.1016/j.semcancer.2008.10.002 PMC2615476

[b34] Offman J. . Defective DNA mismatch repair in acute myeloid leukemia/myelodysplastic syndrome after organ transplantation. Blood 104, 822–828 (2004). 15090454 10.1182/blood-2003-11-3938

[b35] Strangfeld A. . Risk of incident or recurrent malignancies among patients with rheumatoid arthritis exposed to biologic therapy in the German biologics register RABBIT. Arthritis Res. Ther. 12, R5 (2010). 20064207 10.1186/ar2904 PMC2875631

[b36] Kouklakis G. . Development of primary malignant melanoma during treatment with a TNF-α antagonist for severe Crohn’s disease: a case report and review of the hypothetical association between TNF-α blockers and cancer. Drug Des. Devel. Ther. 7, 195–199 (2013). 10.2147/DDDT.S41889 PMC3615922 23569358

[b37] Hudesman D., Lichtiger S. & Sands B. Risk of extraintestinal solid cancer with anti-TNF therapy in adults with inflammatory bowel disease: review of the literature. Inflamm. Bowel Dis. 19, 644–649 (2013). 23314243 10.1097/MIB.0b013e318280ebbd

[b38] Nemes B. . Primary hepatic carcinoid in a renal transplant patient. Pathol. Oncol. Res. 5, 67–69 (1999). 10079384 10.1053/paor.1999.0067

[b39] Colombel J. F. . The safety profile of infliximab in patients with Crohn’s disease: the Mayo clinic experience in 500 patients. Gastroenterology 126, 19–31 (2004). 14699483 10.1053/j.gastro.2003.10.047

[b40] Biancone L. . Infliximab and newly diagnosed neoplasia in Crohn’s disease: a multicentre matched pair study. Gut 55, 228–233 (2006). 16120759 10.1136/gut.2005.075937 PMC1856527

[b41] Peyrin-Biroulet L. . Colon cancer after infliximab therapy for Crohn’s disease in a young patient transplanted for primary sclerosing cholangitis. Am. J. Gastroenterol. 101, 2664–2665 (2006). 17090288 10.1111/j.1572-0241.2006.00809_3.x

