# Supplementary material for: Establishment and characterization of HROC69 – a Crohn´s related colonic carcinoma cell line and its matched patient-derived xenograft
Source: Sci Rep. 2016 Apr 18;6:24671. doi: 10.1038/srep24671 (PMC4834534; doi:10.1038/srep24671)
Supplement: Supplementary Information [file srep24671-s1.pdf]

Supplementary figure 1

Establishment and characterization of HROC69 – a Crohn´s related colonic carcinoma cell line and its matched patient-derived xenograft

Florian Kuehn, Christina S. Mullins, Mathias Krohn, Christine Harnack, Robert Ramer, Oliver H. Krämer, Ernst Klar, Maja Huehns and Michael Linnebacher

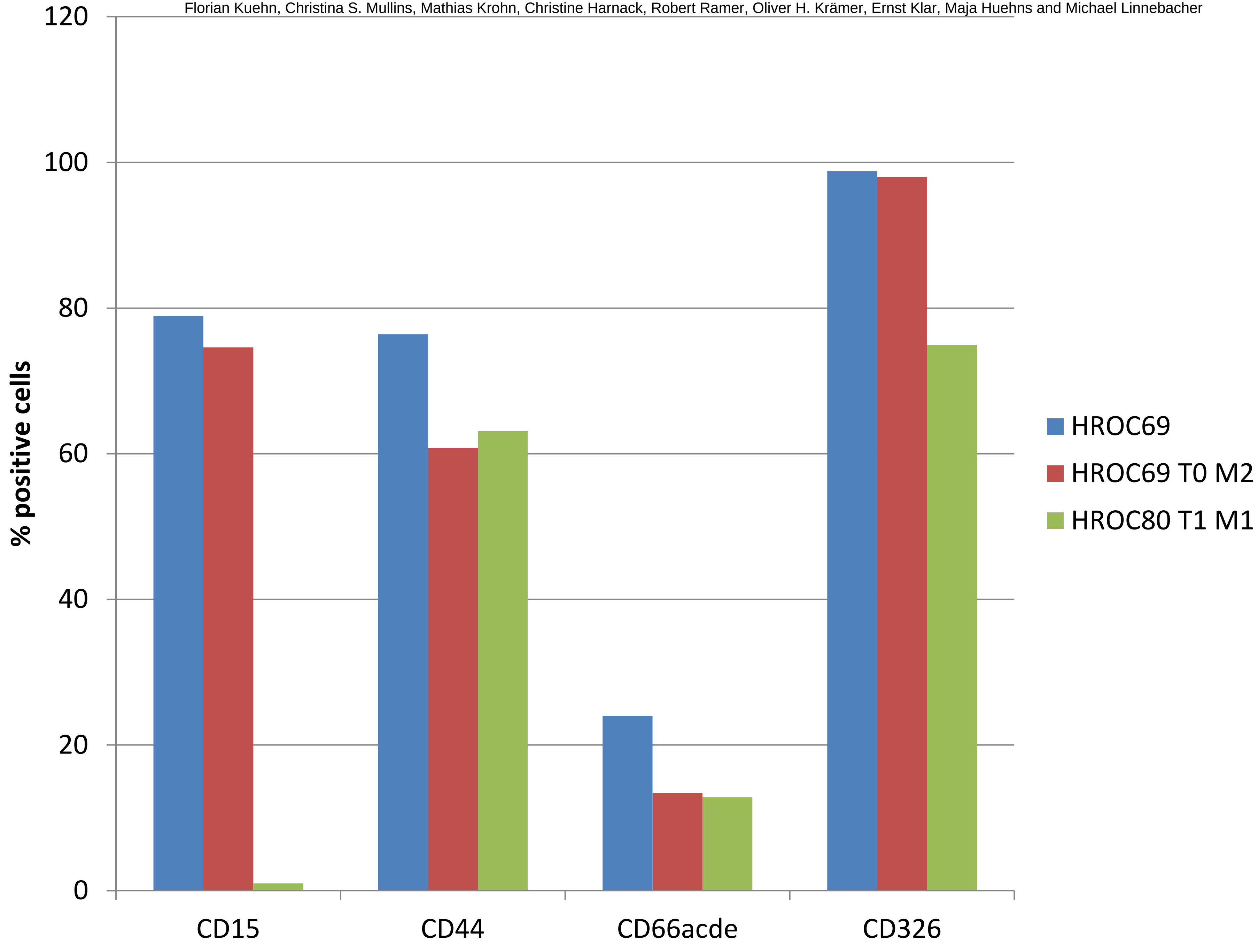

Supplementary figure 2

HROC69 B-LCL

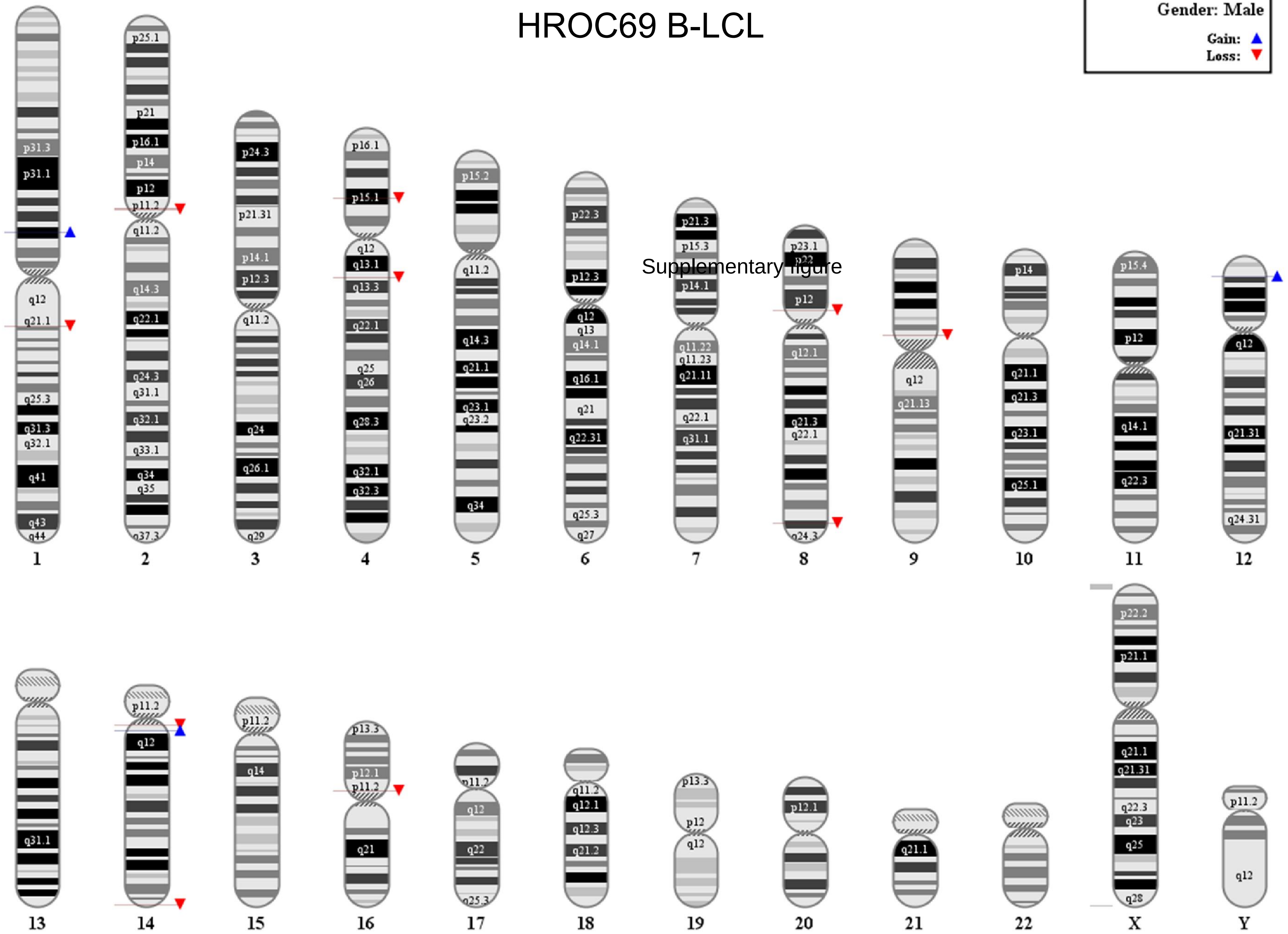

Supplementary figure 2

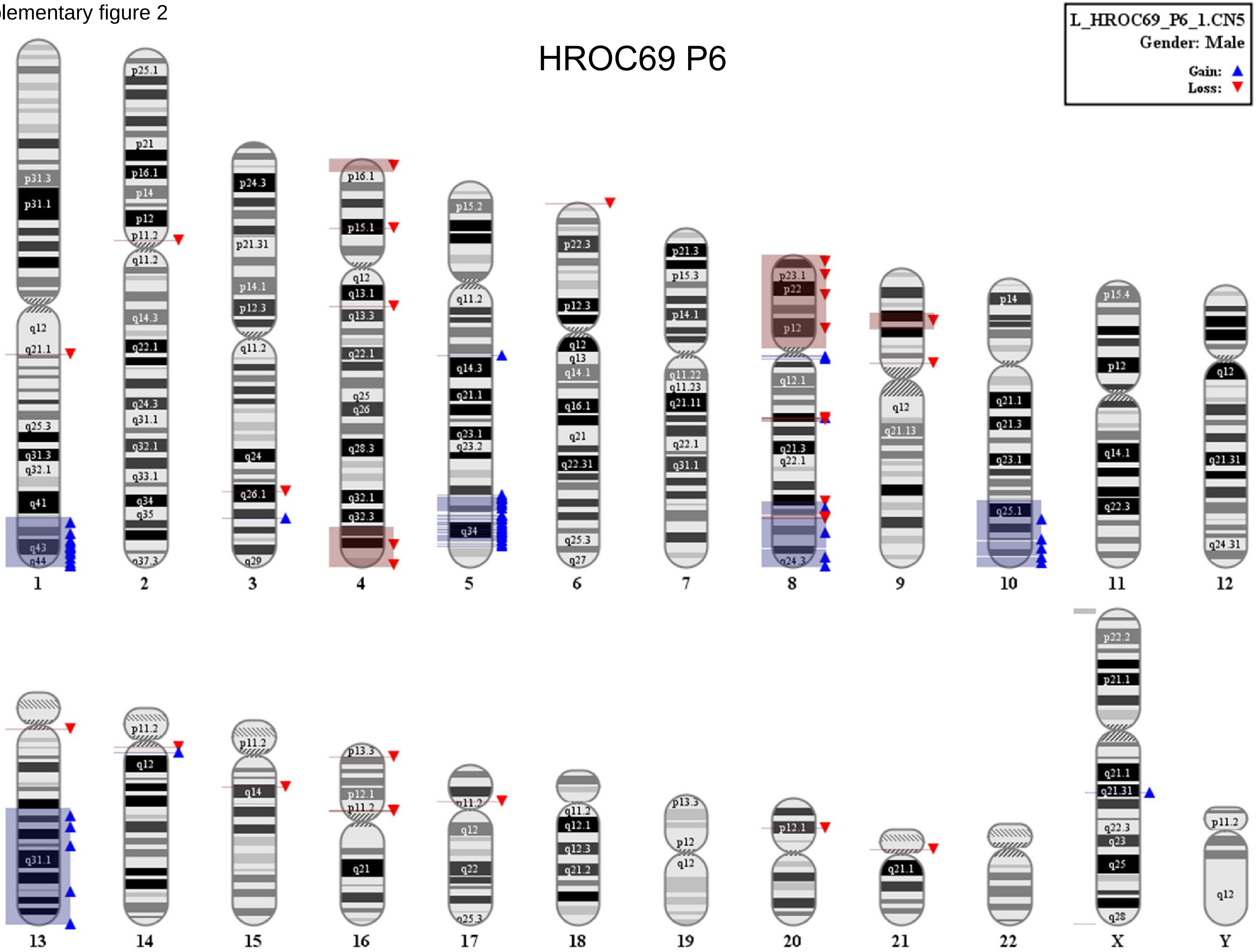

Supplementary figure 2

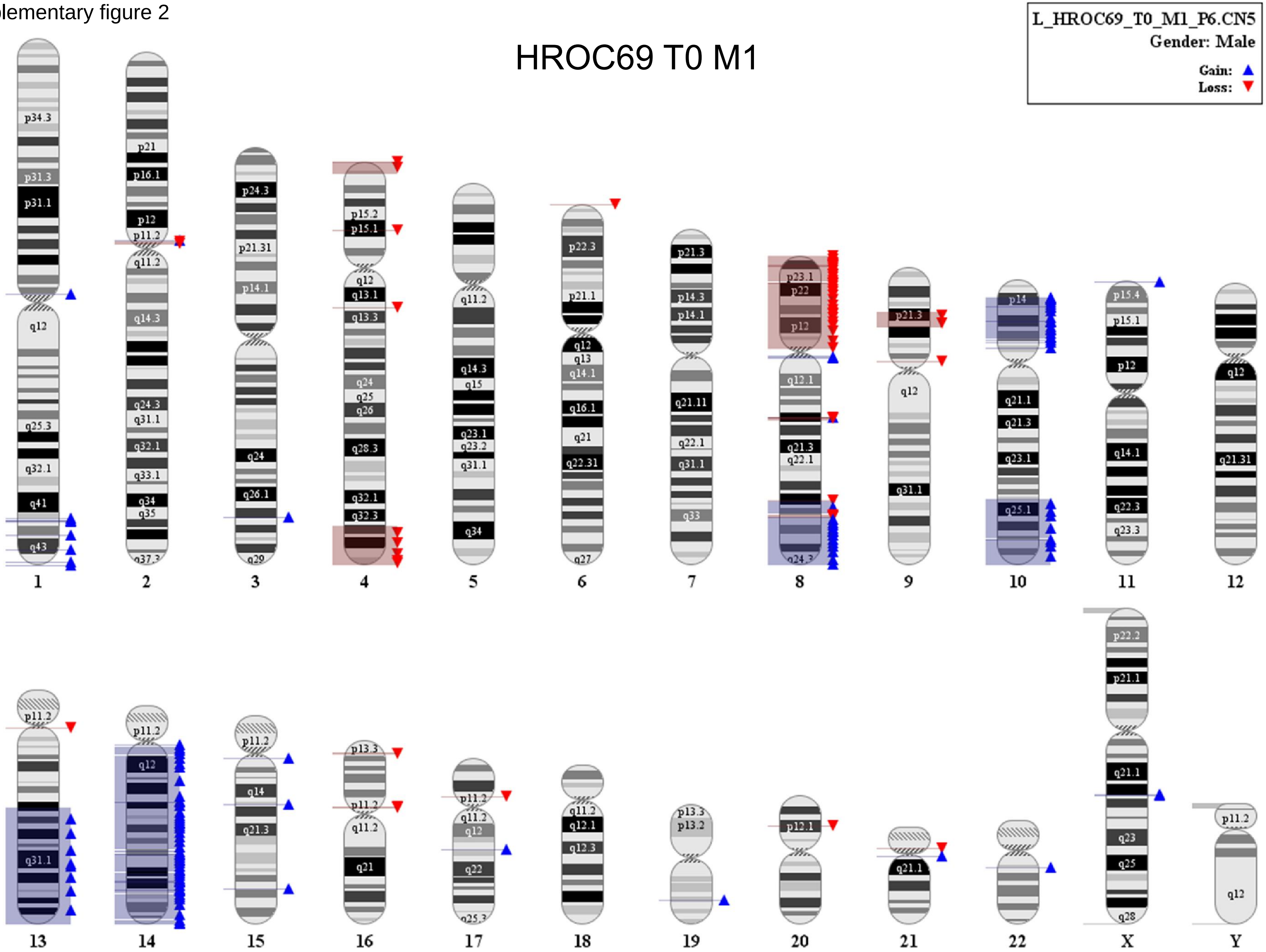

Supplementary figure 3

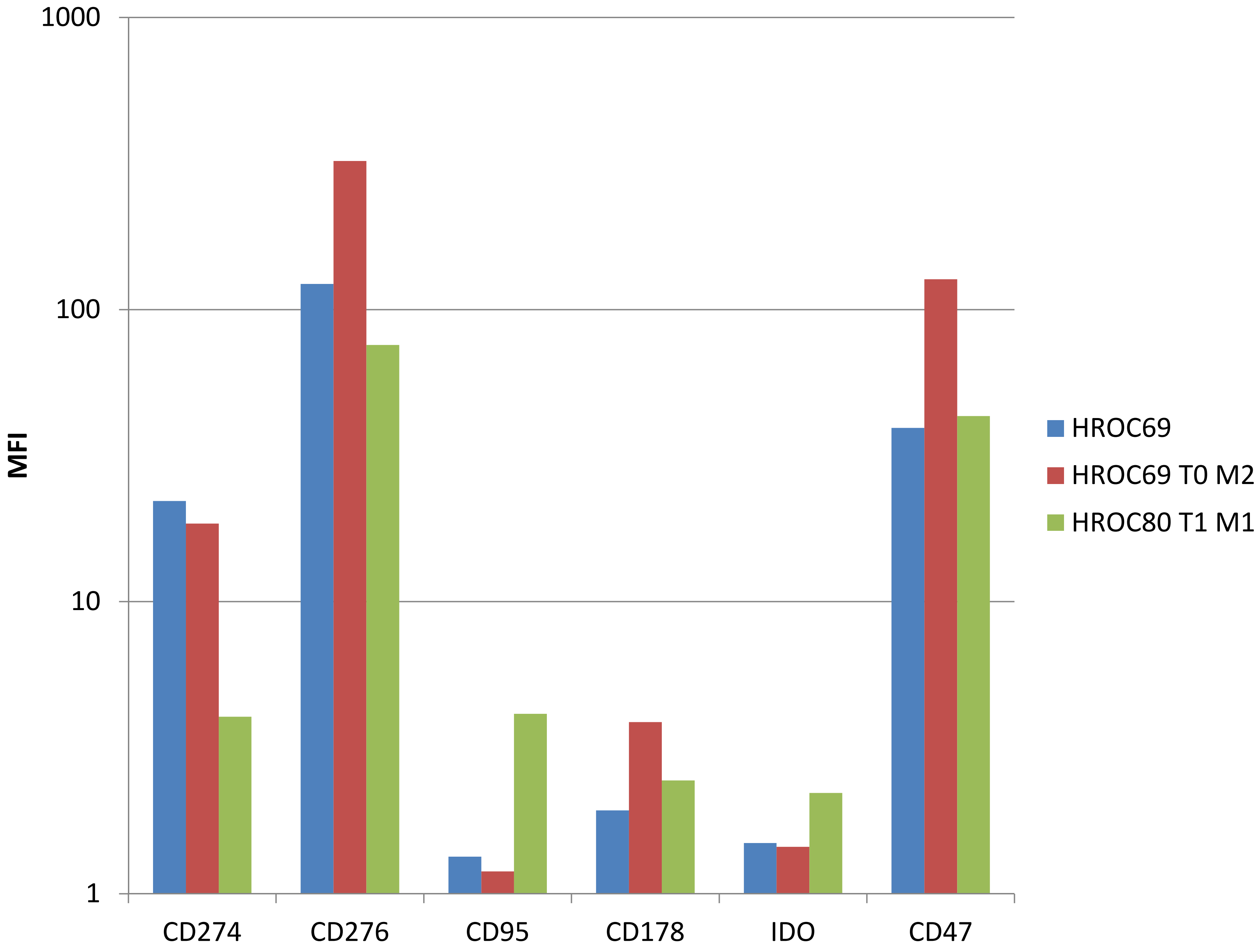

Supplementary table 1

**sample**	**vWA**		**TH01**		**TPOX**		**CSF1 PO**		**D5S818**		**D13S317**	**D7S820**		**D16S539**			**gender**
B-LCL	17	20	7	10	8	12	10	11	12	13	12	n.d.	n.d.	11	12		m
HROC69	17	20	7	10	8	12	10	11	12	13	12	10	12	11	12		m
HROC69 T0 M2	17		7	10	8	12	10	11	12	13	12	10	12	12	13		m
PDX	17	20	7	10	8	12	10	11	12	13	12	10	12	11	12		m

Supplementary table 2

#	fold change	p-value	gene	gene name	function/family	HROC69 up vs normal colon
1	481	0,00021	GAGE5	G antigen 5	cancer-testis antigen	
2	386	0,00030	GAGE2	G antigen 2	cancer-testis antigen	
3	170	0,00057	MAGEA2(B)	melanoma antigen family A2	cancer-testis antigen	
4	148	0,00115	PAGE1	P antigen family, member 1	cancer-testis antigen	
5	137	0,00075	DSCR8	DSCR8	cancer-testis antigen	
6	135	0,00003	HIST1H3I	histone cluster 1	DNA/transcription/translation regulation	
7	126	0,00063	IMP-3	IGF-II mRNA-binding protein 3	DNA/transcription/translation regulation	
8	123	0,00063	GAGE3	G antigen 3	cancer-testis antigen	
9	121	0,00007	GAGE4	G antigen 4	cancer-testis antigen	
10	108	0,00073	CCNK	cyclin K	cell cycle/signaling	
11	106	0,00146	PTPN11	protein tyrosine phosphatase, non-receptor type 11	cell cycle/signaling	
12	103	0,00096	UNQ473	chemokine (C-X-C motif) ligand 17	immune regulation	
13	102	0,00002	SUPT6H	suppressor of Ty 6 homolog	DNA/transcription/translation regulation	
14	101	0,00012	GAGE2	G antigen 2	cancer-testis antigen	
15	100	0,00075	ENTPD6	ectonucleoside triphosphate diphosphohydrolase 6	immune regulation	
16	99	0,00131	SCD	stearoyl-CoA desaturase	metabolism	
17	99	0,00118	TAF13	TATA box binding protein (TBP)-associated factor 13	DNA/transcription/translation regulation	
18	92	0,00014	MAGEA3	melanoma antigen family A3	cancer-testis antigen	
19	87	0,00052	MAGEA6	melanoma antigen family A6	cancer-testis antigen	
20	81	0,00119	HIST1H4L	histone cluster 1	DNA/transcription/translation regulation	
1	-1436	0,00024	SLC26A3	solute carrier family 26	metabolism / tumor suppressor ?	HROC69 down vs normal colon
2	-1317	0,00154	SEPP1	selenoprotein P, plasma, 1	stress response	
3	-1181	0,00028	KRT20	keratin 20, type I	stress response	
4	-1133	0,00074	FABP1	fatty acid binding protein 1	metabolism	
5	-1041	0,00044	CEACAM7	carcinoembryonic antigen-related cell adhesion molecule 7		
6	-816	0,00109	UGT2B17	UDP glucuronosyltransferase 2 family, polypeptide B17	metabolism	
7	-529	0,00078	CA2	carbonic Anhydrase II	metabolism	
8	-515	0,00093	CA1	carbonic Anhydrase I	metabolism	
9	-487	0,00057	PLAC8	placenta-specific 8	cell cycle/signaling	
10	-420	0,00109	ATP1B1	ATP1B1	multiple	
11	-331	0,00012	IGFBP7	insulin-like growth factor binding protein 7	DNA/transcription/translation regulation	
12	-331	0,00092	HPGD	hydroxyprostaglandin dehydrogenase 15-(NAD)	multiple / tumor suppressor	
13	-311	0,00004	PIGR	polymeric immunoglobulin receptor	immune regulation	
14	-309	0,00040	C10orf99	colon-derived SUSD2 binding factor	tumor suppressor	
15	-299	0,00006	C6orf105	androgen-dependent TFPI-regulatingprotein		
16	-245	0,00052	TMEM45B	transmembrane protein 45B		
17	-242	0,00046	LRRC19	leucine rich repeat containing 19		
18	-216	0,00057	SI	sucrase-isomaltase	metabolism	
19	-196	0,00097	TNFSF10	tumor necrosis factor apoptosis-inducing ligand	tumor suppressor	
20	-154	0,001374	HSD17B	hydroxysteroid (17-beta) dehydrogenase 2	metabolism	

Supplementary figure 1: analyses of epithelial markers for CAC and sporadic CRC derived cell lines

Expression of epithelial cell markers (CD15, CD44, CD66acde and CD326) was analysed for the CAC derived cell lines HROC69 (blue) and HROC69 T0 M2 (red) in comparison to the sporadic CRC derived cell line HROC80 T1 M1 (green) by flow cytometry. The percentage of positively stained cells (in comparison to an unstained control) is depicted in the bar chart.

Supplementary figure 2: Genome-Wide Human SNP Array

An overview of chromosomal gains (blue) and losses (red) as detected by SNP 6.0 arrays are given for the patient-derived tumour models (HROC69 cell line and PDX) in comparison to HROC69 Bc as a healthy tissue control.

Supplementary figure 3: analyses of immune checkpoints for CAC and sporadic CRC derived cell lines

Expression of immune checkpoint markers (CD274, CD276, CD95, CD178, IDO and CD47) was analysed for the CAC derived cell lines HROC69 (blue) and HROC69 T0 M2 (red) in comparison to the sporadic CRC derived cell line HROC80 T1 M1 (green) by flow cytometry. Mean fluorescent intensity levels were calculated as follows: (fluorescence intensity of antibody staining - fluorescence intensity of control)/ fluorescence intensity of control).

Supplementary table 1: Identity check

Nine classical loci were used in a Short Tandem Repeat analysis to check the identity of the tumour models. The alleles of the loci are displayed for the patient-

derived cell line HROC69, the PDX-derived cell line HROC69 T0 M2, the PDX and HROC69 Bc as normal tissue control.

Supplementary table 2: Whole human genome expression array

The 20 most up and down regulated genes of the patient-derived cell line HROC69 in comparison to expression in normal colon tissue are listed. Information on the fold change, p-value (ANOVA), gene symbol, gene name and gene function are summarized.
